# The LuxS Based Quorum Sensing Governs Lactose Induced Biofilm Formation by *Bacillus subtilis*

**DOI:** 10.3389/fmicb.2015.01517

**Published:** 2016-01-08

**Authors:** Danielle Duanis-Assaf, Doron Steinberg, Yunrong Chai, Moshe Shemesh

**Affiliations:** ^1^Department of Food Quality and Safety, Institute for Postharvest Technology and Food Sciences, Agricultural Research Organization, The Volcani Center Bet-Dagan, Israel; ^2^Biofilm Research Laboratory, Institute of Dental Sciences, Faculty of Dental Medicine, Hebrew University Hadassah Medical SchoolJerusalem, Israel; ^3^Department of Biology, Northeastern University, BostonMA, USA

**Keywords:** *B. subtilis*, biofilm, lactose, quorum sensing, AI-2 LuxS system

## Abstract

*Bacillus* species present a major concern in the dairy industry as they can form biofilms in pipelines and on surfaces of equipment and machinery used in the entire line of production. These biofilms represent a continuous hygienic problem and can lead to serious economic losses due to food spoilage and equipment impairment. Biofilm formation by *Bacillus subtilis* is apparently dependent on LuxS quorum sensing (QS) by Autoinducer-2 (AI-2). However, the link between sensing environmental cues and AI-2 induced biofilm formation remains largely unknown. The aim of this study is to investigate the role of lactose, the primary sugar in milk, on biofilm formation by *B. subtilis* and its possible link to QS processes. Our phenotypic analysis shows that lactose induces formation of biofilm bundles as well as formation of colony type biofilm. Furthermore, using reporter strain assays, we observed an increase in AI-2 production by *B. subtilis* in response to lactose in a dose dependent manner. Moreover, we found that expression of *eps* and *tapA* operons, responsible for extracellular matrix synthesis in *B*. *subtilis*, were notably up-regulated in response to lactose. Importantly, we also observed that LuxS is essential for *B. subtilis* biofilm formation in the presence of lactose. Overall, our results suggest that lactose may induce biofilm formation by *B. subtilis* through the LuxS pathway.

## Introduction

Bacteria often use quorum sensing (QS) as cell–cell communication mechanism to control expression of genes that affect a variety of cellular processes (Fuqua et al., 1994; Miller and Bassler, 2001; Bai and Rai, 2011). QS is based on production, secretion and response to small signaling molecules, termed autoinducers (AI; Bai and Rai, 2011). AI-2, a furanosyl-borate-diester (Chen et al., 2002) is referred as a “universal autoinducer” as it is found in numerous Gram positive and Gram negative bacteria (Schauder and Bassler, 2001; Xavier and Bassler, 2003). AI-2 is synthesized by LuxS through steps involving conversion of ribose-homocysteine into homocysteine and 4,5-dihydroxy-2,3pentanedione (DPD), a compound that cyclizes into several furanones in the presence of water (Schauder et al., 2001). QS modulates various cellular processes involved mainly in the regulation of virulence factors, sporulation, motility, toxin production (Hammer and Bassler, 2003; Henke and Bassler, 2004; Smith et al., 2004; Waters and Bassler, 2006) and formation of a structured multicellular community of bacterial cells, also termed biofilm (Hall-Stoodley et al., 2004; Kolter and Greenberg, 2006). It appears that biofilm formation is the most successful strategy for bacteria to survive unfavorable environmental conditions (Stewart and Costerton, 2001; Hall-Stoodley et al., 2004). Bacteria in biofilms are highly resistant to disinfection and antibiotic treatments, therefore biofilm formation is considered as a major problem in the industrial fields and in medicine (Simoes et al., 2010).

*Bacillus subtilis* is a Gram-positive non-pathogenic bacterium, which is a facile model microorganism for biofilm research. *B. subtilis* possesses the ability to form different types of biofilms in different environmental conditions: colony type biofilm at solid-air interface, pellicle at liquid–air interface as well as submerged biofilm at solid-liquid interface (Vlamakis et al., 2013). *B. subtilis* cells can sense different environmental and physiological signals, which may activate one of its histidine sensor kinases. Those kinases are responsible for phosphorylation of Spo0A, a master regulator in the cell. Phosphorylated Spo0A leads to down-regulation of the transcriptional repressors AbrB and SinR, which keeps expression of genes for production of extracellular matrix turned off when conditions are not propitious for biofilm growth (Branda et al., 2006; Vlamakis et al., 2013). When a signal is introduced for biofilm formation, *B. subtilis* cells are shifted from motile bacteria to bacterial chains that stick together by producing an extracellular matrix (Branda et al., 2001; Kobayashi, 2007). The matrix has an important role during the biofilm formation. It provides an attaching source for other bacteria in the surrounding environment and therefore plays a crucial step in biofilm progression (Branda et al., 2001; Kobayashi, 2007). The matrix consisted of two main components, an extracellular polysaccharide (EPS) synthesized by the products of the *epsA-O* operon, and amyloid fibers encoded by *tasA* located in the *tapA-sipW-tasA* operon (Branda et al., 2006; Vlamakis et al., 2013).

Biofilms formed by *Bacillus* species are vastly found throughout dairy processing plants (Oosthuizen et al., 2001). Moreover, the major source of contamination of dairy products is often associated with members of the *Bacillus* genus (Sharma and Anand, 2002; Simoes et al., 2010). Recently, it was found that certain milk components enhance biofilm formation by *Bacillus* species (Pasvolsky et al., 2014). Lactose, a β1,4-linked disaccharide, is the main carbohydrate in milk and numerous dairy products. Our previous study showed that lactose increases biofilm formation by the Gram-positive bacteria *Streptococcus mutans* (Assaf et al., 2015). Since lactose is an abundant disaccharide sugar in milk and its products, it might serve as an environmental trigger for biofilm formation by other bacteria too, for instance *B. subtilis*. Interestingly, it has been shown that *B. subtilis* might use QS to regulate motility and biofilm formation (Lombardía et al., 2006). However, the link between sensing environmental cues and the QS induced biofilm formation by *B. subtilis* is poorly known. Therefore, the aim of this study was to investigate the role of lactose, the primary sugar in milk, on biofilm formation by *B. subtilis* and its possible link to QS process.

## Materials and Methods

### Strains and Growth Media

Strains used in this study are listed in **Table 1**. For routine growth, all bacterial strains were grown in Lysogeny broth (LB; 10 g of tryptone (Neogen, Lansing, Michigan, USA), 5 g of yeast extract (Neogen, Lansing, MI, USA) and 5 g of NaCl per liter) and incubated at 37°C at 150 rpm for 5 h. The LB medium was solidified by addition of 1.5% agar (Neogen, Lansing, MI, USA) (Pasvolsky et al., 2014). Although, LB is suitable for bundle formation experiments, it was found to be less favorable for colony type biofilm or pellicle formation (Branda et al., 2001; Shemesh and Chai, 2013). Therefore, we studied colony biofilm and pellicle formation using chemically defined medium (CDM). CDM was prepared as previously described with slight modifications (Branda et al., 2001). Briefly, CDM contained 5mM potassium phosphate (pH 7), 100 mM 3-[*N*-Morpholino] propane sulfonic acid (MOPS) (pH 7), 2 mM MgCl_2_, 700 μM CaCl_2_, 50 μM MnCl_2_, 50 μM FeSO_4_, 1 μM ZnCl_2_, 2 μM thiamine (Sigma–Aldrich, St. Louise, MI, USA), 0.5% glycerol, 0.5% glutamate, 50 μg/ml tryptophan (Sigma–Aldrich, St. Louise, MI, USA), and 50 μg/ml phenylalanine. (Sigma–Aldrich, St. Louise, MI, USA). For CDA, 1.5% agar (Neogen, Lansing, MI, USA) was added. Medium and plates were freshly prepared and used the following day.

**Table 1:** Strains used in this study.

Strain	Genotype	Characteristic description	Reference
***Bacillus subtilis***			
NCIB3610	wild type	Undomesticated WT strain	Branda et al., 2001
YC161	P*_spank_-gfp*	Produces GFP constantly	Chai et al., 2011
YC164	P*_eps_-gfp*	Produces GFP under the control of promoter for *eps*	Chai et al., 2008
YC189	P*_tapA_-cfp* at the *amy*E locus in 3610, Spec^R^	Produces CFP under the control of promoter for *tapA*	Chai et al., 2008, Pasvolsky et al., 2014
	*ΔluxS*	Mutant in *luxS* gene Which does not produce AI-2	Chai lab collection
RL3852	*ΔepsH* in 3610, Tet^R^	Mutant in production of EPS	Kearns et al., 2005
SB505	*ΔtasA* in 3610, Spec^R^	Mutant in production of amyloid fibers	A gift of Branda S.
***V. harveyi***			
MM77	*ΔluxLM*, Tn5, *ΔluxS*, Cm^R^	Mutant in both systems of quorum sensing (QS) which does not produce AI-1 and AI-2	Surette et al., 1999


LBGM media was prepared as described previously by supplementing LB with 1% (v/v) glycerol and 0.1 mM MnSO_4_ (Shemesh and Chai, 2013).

### Lactose Preparation

A stock 50% lactose (w/v) (J. T. Baker, London, UK) solution was prepared in distilled deionized water and sterilized using a 0.2 μm filter (Whatman, Dassel, Germany). The stock solution of lactose was diluted in LB to final concentrations of 0–5% (w/v) or CDA to final concentration of 3% (w/v) (Assaf et al., 2015).

### Biofilm Formation

Colony biofilms are produced when cells are placed on a solid agar surface. Importantly, one of the major characteristics of biofilm colony is the production of extracellular matrix which harbors the biofilm bacteria (Vlamakis et al., 2013). For colony type biofilm formation (Branda et al., 2001), starter cultures were prepared as describe above. 2.5 μl (around 3 × 10^5^ CFU) from the starter culture was dropped on CDA with or without 3% lactose. The plates were incubated at 30°C for 24 h. Images were taken using a Zeiss Stemi 2000-C microscope with an axiocamERc 5s camera.

For bundle formation, an overnight culture of cells was diluted 1:100 (to obtain O.D._(600)_ of 0.07) into LB supplemented with or without 3% lactose (w/v). The samples were incubated at 37°C at 50 rpm for 5 h (O.D._(600)_ of 1). One milliliter of each sample was collected and centrifuged at 5000 rpm for 2 min. The supernatant was discarded, the pellet was re-suspended and 3 μl of the suspension placed on a glass slide was visualized in a transmitted light microscope using Nomarski differential interference contrast (DIC), at 40× magnification (Pasvolsky et al., 2014; Oknin et al., 2015). Furthermore, a confocal laser scanning microscope (CLSM) was used to visualize cyan fluorescent protein (CFP) or green fluorescent protein (GFP) expression. CFP expression of strain YC189 was observed using 458-nm argon laser, while GFP expression of strains YC161 and YC164 was observed using 488-nm argon laser (Zeiss LSM510 CLS microscope, Carl Zeiss, Oberkochen, Germany).

For pellicle formation, bacteria were inoculated from the agar plates into LB broth and incubated for 5 h at 37°C at 150 rpm. Next, 5 μl of the culture was seeded in a 12 wells plate (Nunc, Roskild, Denmark) containing 4 ml of CDM per well. The plates were incubated at 30°C. Pictures were taken after 24 h using SAMSUNG Galaxy camera.

### AI-2 Production Assay

To determine the effect of lactose on AI-2 production, we used a bioluminescence assay as described before (Aharoni et al., 2008; Shemesh et al., 2010). Briefly, *B. subtilis* cells were grown in conditions inducing bundle formation as described above. One milliliter of each sample was collected and centrifuged at 5000 rpm for 2 min. Supernatant was collected and neutralized to pH 7 using 1 M NaOH. An overnight culture of *Vibrio harveyi* MM77, a mutant strain which does not produce either AI-1 nor AI-2, was diluted 1:5,000 in a mixture of 90% (v/v) fresh AB medium and 10% (v/v) neutralized supernatant to a total volume of 200 μl per well. The negative control contained bacteria in fresh AB medium alone, while the positive control contained the bacteria, fresh AB medium and 10% (v/v) supernatant medium containing AI-2 of *V. harveyi* BB152 (AI-1–, AI-2+). The luminescence readings were performed in triplicate in white 96-well plates with an optic bottom (Nunc, Roskild, Denmark) using a plate reader (GENiosTECAN, NEOTEC Scientific Instrumentation Ltd. Camspec, Cambridge, UK) at 30°C. Luminescence measurements were recorded every 30 min in parallel with optical density (595 nm) readings. To avoid dissimilarities caused by differences in growth rates, the relative luminescence (RLU) was calculated. Briefly, the value of each reading was divided by the optical density values to normalize the luminescence value of each sample to its cell density. Fold induction above the non-specific luminescence background of the negative control was determined at the end of bacterial growth, after approximately 20 h of growth. The area under the curve (AUC) was calculated to better demonstrate the differential expression in RLU values by means of the sum of: the average of *Y* values/the average of *X* values (Aharoni et al., 2008; Soni et al., 2015).

### AI-2 Effect on Biofilm Formation

To determine the effect of AI-2 on bundle formation as well as *tapA* expression, we used (S)-4,5-Dihydroxy-2,3-pentandione (DPD) (Omm Scientific, Inc, Dallas, TX, USA) which is the precursor for AI-2. Bacterial cells prepared as described above and were incubated in the presence of DPD in LB at 37°C at 50 rpm for 5 h. The cells were collected and visualized in a transmitted light microscope using DIC. Furthermore, a CLSM was used to visualize CFP expression using 458-nm argon laser (Oknin et al., 2015). For complementation tests, DPD was supplemented in LB medium to final concentration of 200 μM as an exogenous precursor for AI-2.

### Statistical Analysis

The data obtained were analyzed statistically by means of ANOVA following *post hoc t*-test with Bonferroni correction using Microsoft Excel software. *P*-values less than 0.01 were considered significant.

## Results

### Lactose Induces Biofilm Formation by *B. subtilis*

**FIGURE 1:**
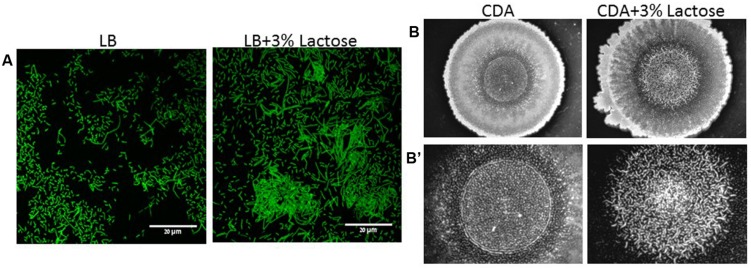
**Lactose induces biofilm formation by *B. subtilis.*** **(A)** CLSM images of bundles formation. Overnight cultures of *B. subtilis* (YC161) were diluted into LB or LB supplemented with 3% lactose. Cultures were then incubated for 5 h at 37°C and 50 rpm. A sample from each culture was then analyzed using a confocal microscope. Images are representative of three biological repeats. **(B)** Colony biofilm was generated on chemical defined agar (CDA) and CDA supplemented with 3% lactose. **(B’)** Zoomed images of the center of generated biofilm. The pictures were taken using a Zeiss Stemi 2000-C microscope with an axiocamERc 5s camera. Images are representative of four biological repeats.

Initially, we found that addition of lactose to growth media such as LB or chemical defined agar (CDA) enhances biofilm formation by *B. subtilis*. As it can be seen in **Figure 1A**, a majority of *B. subtilis* (YC161) cells preferably generated long chains of cells attaching to each other to form a biofilm-related structure (bundle) in the presence of lactose. Similarly, lactose also induced colony type biofilm formation on CDA, as seen in the center of the colony (**Figure 1B**). The structure of the biofilm formed on the CDA with addition of lactose has higher structure complexity. Accordingly, the morphology of the biofilm in the presence of lactose is more developed and structured as seen in the center of the colony (**Figure 1B**). Subsequently, we tested whether the increase in biofilm formation in the presence of lactose is due to the increase in bacterial growth rate. The bacterial growth of *B. subtilis* was not affected by addition of lactose (**Supplementary Figure S1**). Therefore, we assume that the effect of lactose is specific to the biofilm formation.

### Lactose Up-Regulates Expression of Genes Associated with Extracellular Matrix Production

**FIGURE 2:**
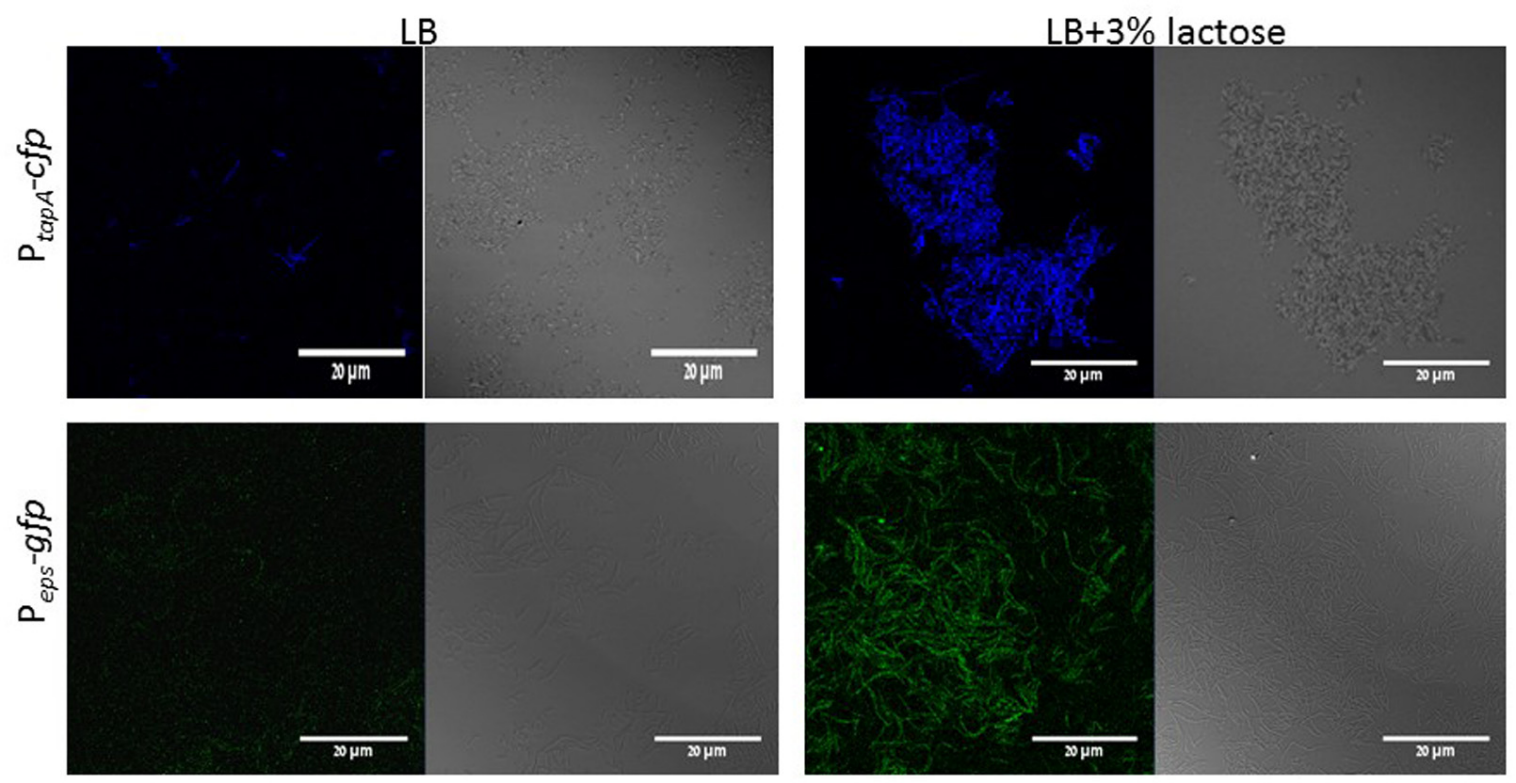
**Lactose triggers expression of *tapA* and *eps* operons in *B. subtilis*.** WT cells harboring P*_tapA_-cfp* (YC189) or P*_eps_-gfp* (YC164) were grown in LB or LB supplemented with 3% lactose. Cultures were then incubated for 5 h at 37°C and 50 rpm. A sample from each culture was then analyzed using a confocal microscope. The right picture are the bacteria taken using Nomarski differential interference contrast (DIC), at 40× magnification and the left pictures are the fluorescent bacteria. The top panel shows the expression of CFP and the bottom panel expression of GFP. Images are representative of five biological repeats.

**FIGURE 3:**
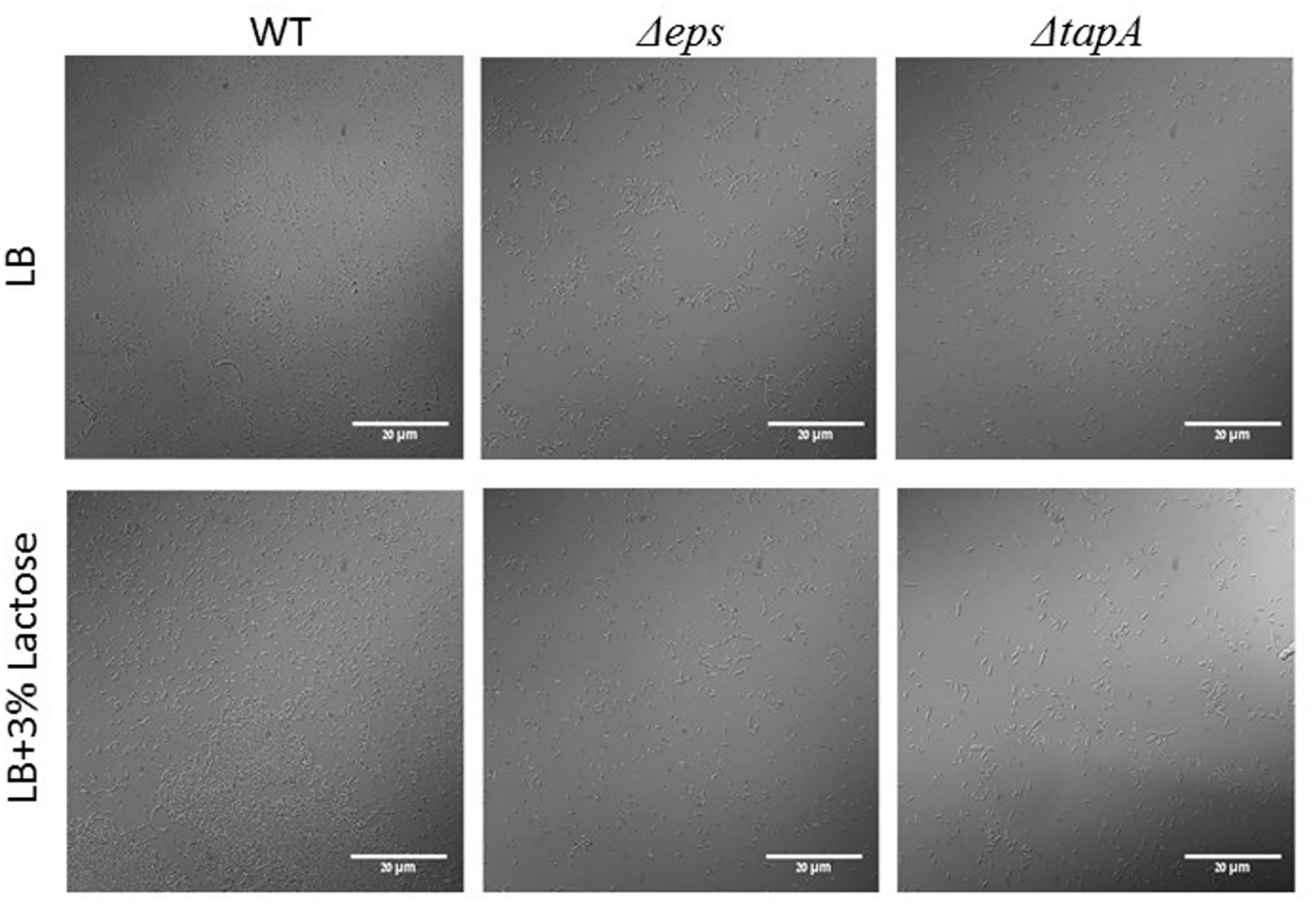
**The *epsA-O* and *tapA-sipW-tasA* operons are essential for bundle formation in the presence of lactose.** The WT, *ΔepsH* and *ΔtasA* cells of *B. subtilis* were diluted into LB or LB supplemented with lactose. Cultures were incubated for 5 h at 37°C and 50 rpm. A sample from each culture was analyzed using a confocal microscope. Images are representative of two biological repeats.

In order to confirm our above findings and to determine if the bundles induced by lactose are biofilm related, we used genetically modified *B. subtilis* strains, which express fluorescent proteins under the control of important extracellular matrix related promoters. To examine the expression of *tapA* operon, we used the strain (YC189) which produces CFP under the control of the *tapA* promoter, whereas, the expression of *eps* operon was determined using strain (YC164) which produces GFP under the control of *eps* promoter (Chai et al., 2008). The amounts of the fluorescent proteins as well as their intensity represent the expression of the tested promoter in the different samples. As it is demonstrated in **Figure 2**, the expression of both operons was increased as a result of lactose introduction into the growth medium. Moreover, mutant strains which are defected in production of extracellular matrix showed deficiency in bundles formation in the presence of lactose (**Figure 3**).

### Lactose Triggers AI-2 Production

**FIGURE 4:**
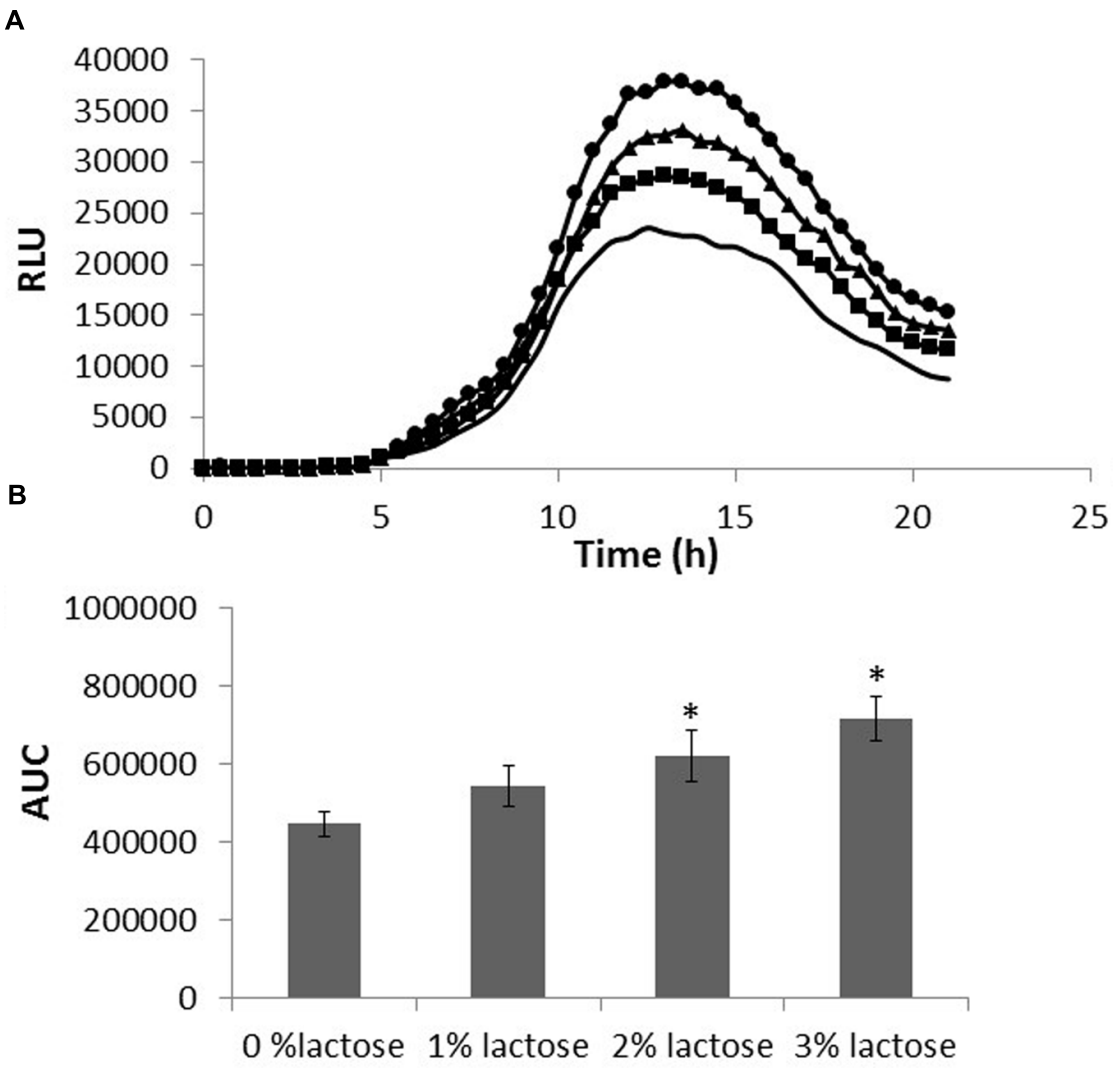
**Lactose induces AI-2 secretion in *B. subtilis.*** *B. subtilis* cells were grown in LB supplemented with 0–3% lactose. Cultures were then incubated for 5 h at 37°C and 50 rpm. A supernatant sample from each culture was taken and incubated with *Vibrio harveyi* MM77. Optical density and luminescence data were recorded every 30 min. **(A)** Bioluminescence kinetics using relative luminescence (RLU). The data are displayed as a mean value of results from two biological repeats each performed as triplicate. (-LB, 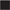
1% lactose, 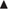
2% lactose, ●3% lactose). **(B)** The area under the curve (AUC). The data were analyzed by means of ANOVA following *post hoc t*-test with Bonferroni correction. ^∗^*P*-value < 0.01 compared to control.

Next, we decided to test whether lactose affects AI-2 production. Using *V. harveyi* MM77 as a reporter strain enables us to examine the effect of lactose on QS via the LuxS dependent pathway. Supernatants from *B. subtilis*, grown with or without lactose, were used for evaluating the amount of AI-2 secreted to the media. The RLU indicates the relative amount of AI-2 in the suspension; a peak of the relative bioluminescence was detected following 14 h in all tested samples which was found to be remarkably higher in the presence of lactose (in dose dependent manners; **Figure 4A**). Indeed, there was a significantly increase in the production of AI-2 by *B. subtilis* cells in the presence of all tested lactose concentrations especially in the presence of 3% of lactose (**Figure 4B**).

### *luxS* is Essential for Biofilm Formation in the Presence of Lactose

**FIGURE 5:**
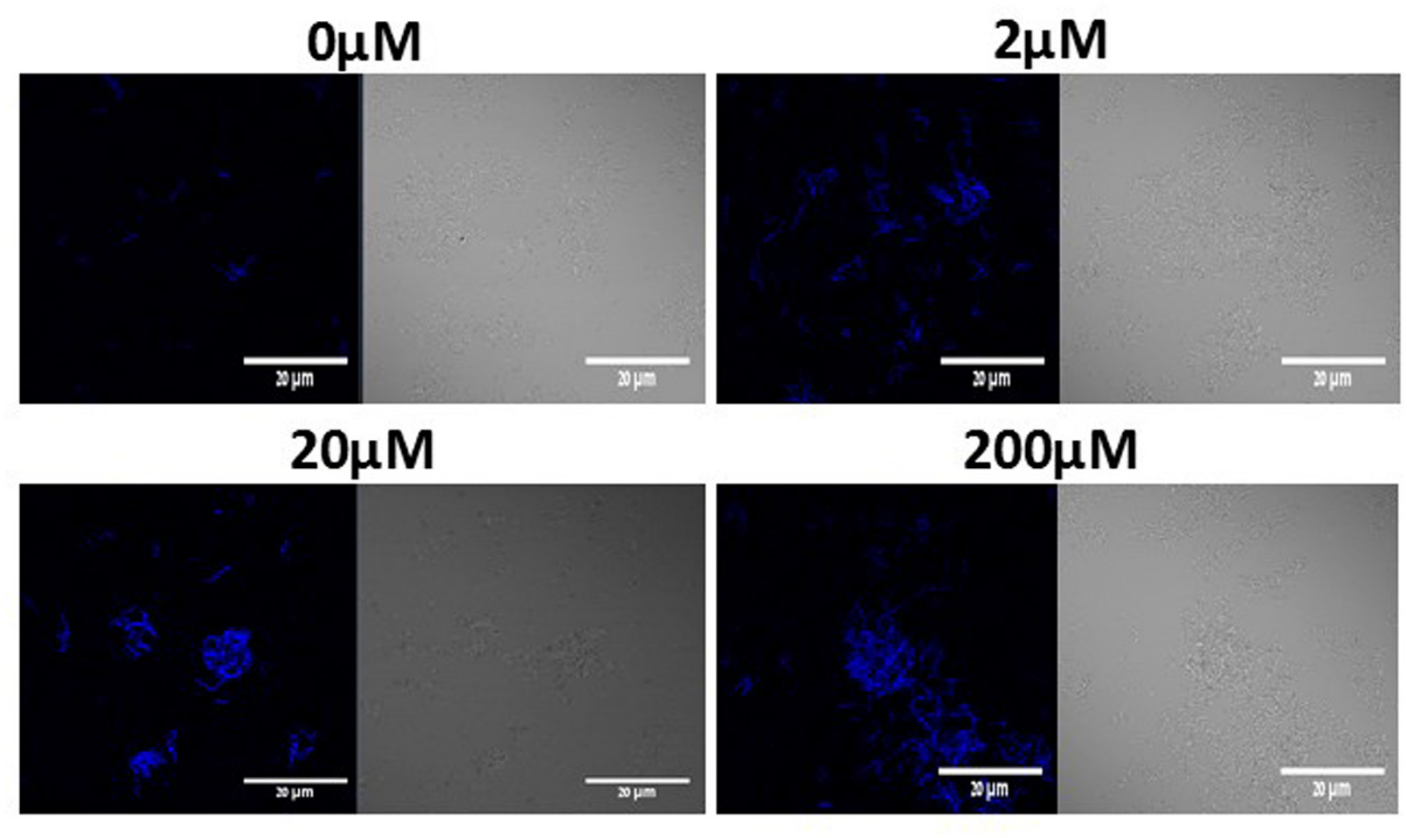
**DPD triggers expression of *tapA* operon and bundle formation in *B. subtilis.*** WT cells harboring P*_tapA_-cfp* were grown in LB or LB supplemented with 0–200 μM DPD. Cultures were then incubated for 5 h at 37°C and 50 rpm. A sample from each culture was then analyzed using a confocal microscope. The right pictures are the bacteria taken using DIC, at 40× magnification and the left pictures are the fluorescent bacteria. Images are representative of three biological repeats.

We further investigated the connection between LuxS dependent QS and induction in biofilm formation. Thus, we used the YC189 strain (harboring the P*_tapA_-cfp* transcriptional fusion) which was grown in the presence of different concentrations of DPD (precursor for AI-2). Interestingly, increasing concentrations of DPD stimulated the biofilm bundles formation as well as *tapA* expression (**Figure 5**). The induction in bundle formation and *tapA* expression seems to be in linear correlation with the concentration of DPD.

**FIGURE 6:**
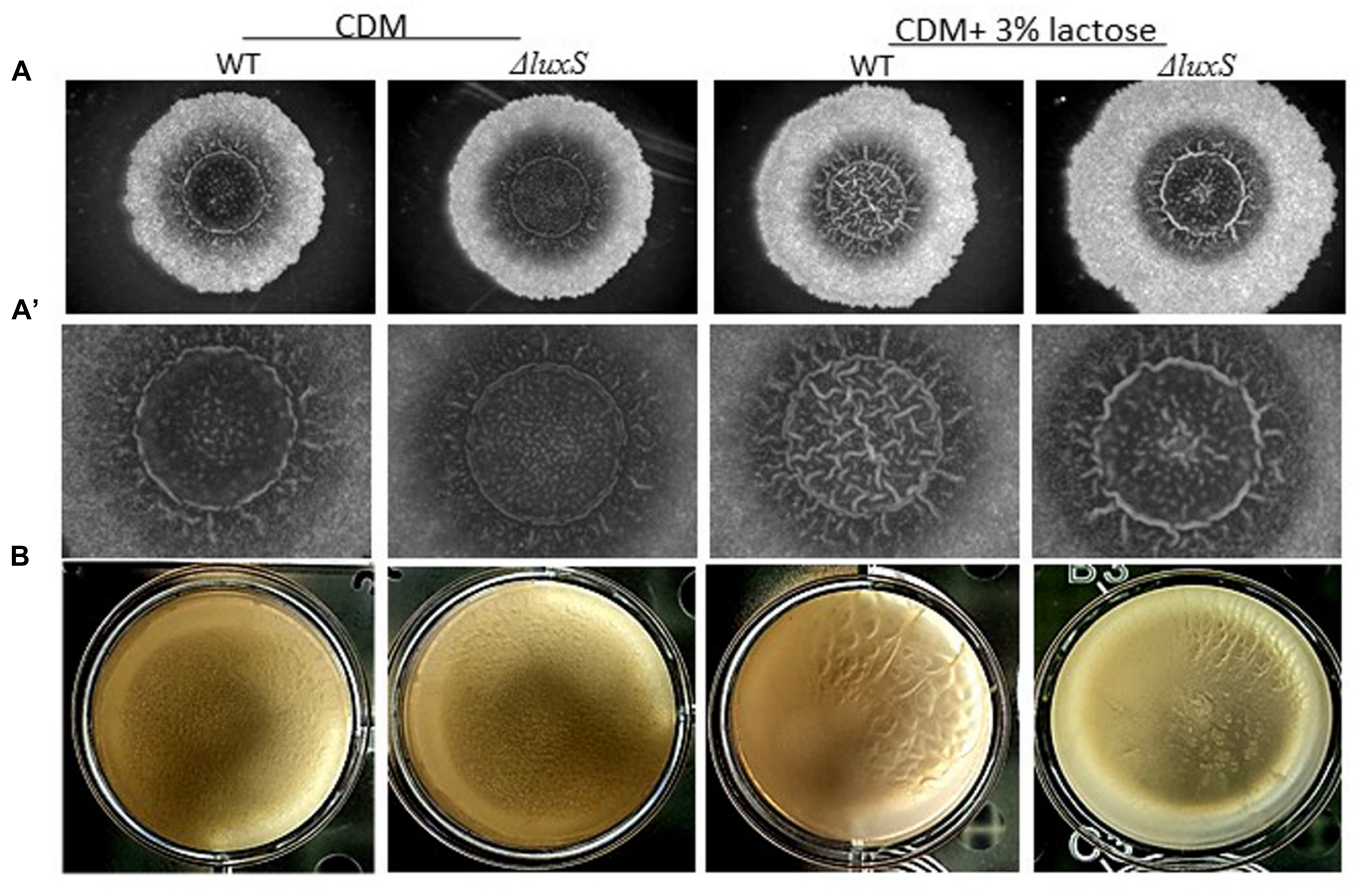
***luxS* is essential for colony biofilm formation in the presence of lactose.** **(A)** WT and *ΔluxS* cells were used for colony biofilm formation. Biofilms were generated on chemically defined agar (CDA) and CDA supplemented with 3% lactose. **(A’)** are zoomed images of the center of generated biofilm. The pictures were taken using a Zeiss Stemi 2000-C microscope with an axiocamERc 5s camera. Images are representative of four biological repeats. **(B)** WT and *ΔluxS* cells were used for pellicle biofilm formation. Biofilms were generated in chemical defined medium (CDM) and CDM supplemented with 3% lactose. Pictures were taken using Sumsung galaxy digital camera. Images are representative of two biological repeats.

**FIGURE 7:**
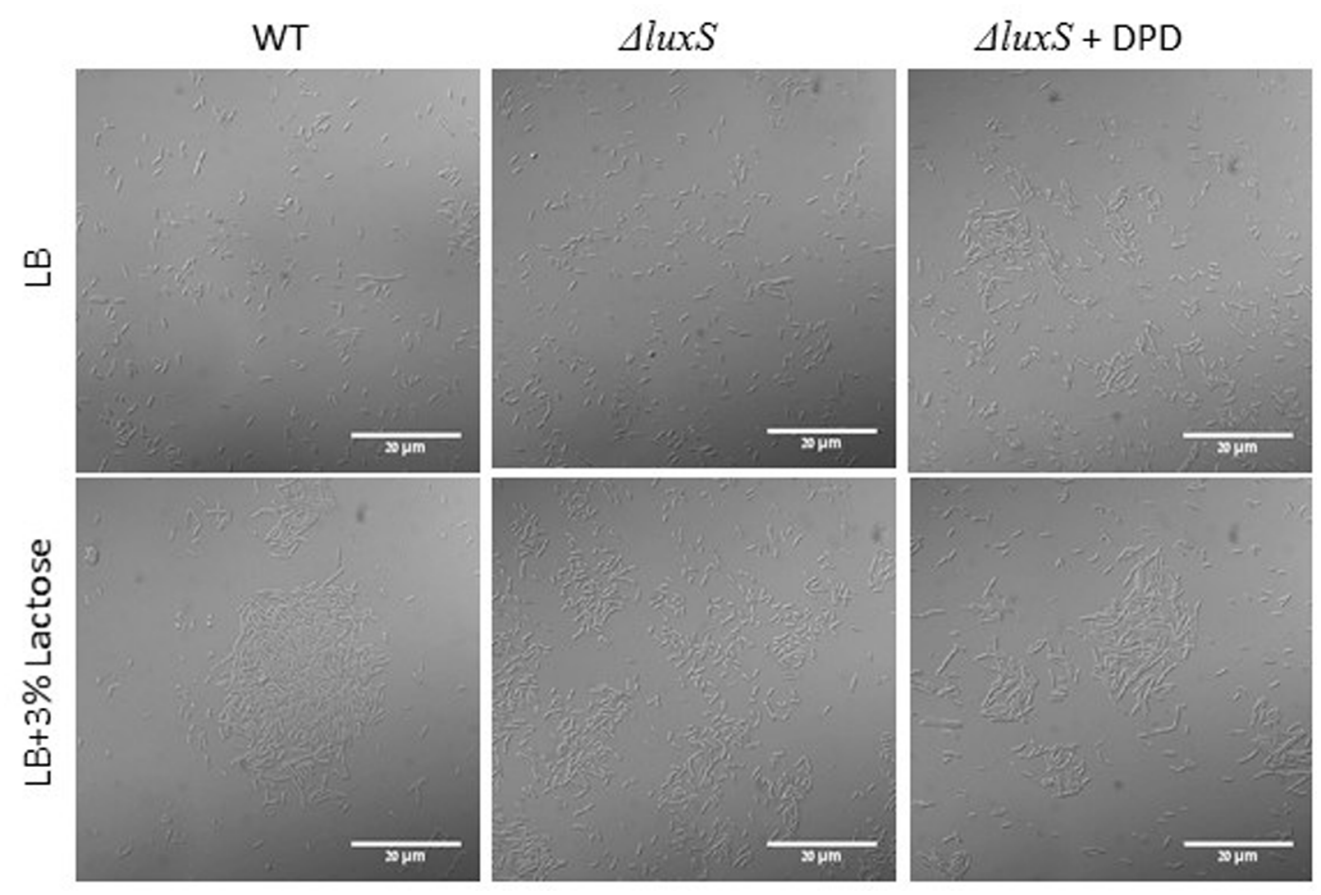
**LuxS is essential for biofilm bundle formation in the presence of lactose.** WT and *ΔluxS* cells were diluted into LB or LB supplemented with lactose. For complementation tests, 200 μM DPD was added to suspensions containing mutant cells. Cultures were then incubated for 5 h at 37°C at 50 rpm. Samples of each culture were then analyzed using a confocal microscope. Images are representative of four biological repeats.

To further investigate a possible role of LuxS on biofilm formation in the presence of lactose, we tested the ability of *B. subtilis luxS* mutant to form bundle as well as pellicle and colony biofilm with or without lactose. As seen in **Figure 6**, the *ΔluxS* mutant is somehow defected in generating developed and structured pellicle and colony biofilm in the presence of lactose compared to the WT. Furthermore, *ΔluxS* mutant could not form biofilm bundles in the presence of lactose (**Figure 7**). Interestingly, addition of DPD restored at least partially the bundling phenotype of the *ΔluxS* mutant (**Figure 7**).

## Discussion

Our results show that lactose triggers bundle formation as well as formation of colony type biofilm by *B. subtilis.* This result falls in line with our previous study which showed that lactose enhances biofilm formation by *Streptococcus mutans* (Assaf et al., 2015). Expression of *epsA-O* and *tapA* operons, which are responsible for biofilm matrix production, were notably increased when lactose was added to the LB medium (**Figure 2**). Interestingly, induction in expression of both operons is correlated with biofilm bundles formation by *B. subtilis* cells. Bundle formation is one of the first stages in biofilm development (Branda et al., 2001). Moreover, investigation of the mutant strains for these operons shows absence of the bundling phenotype as a response to lactose (**Figure 3**). This result indicates that lactose induce biofilm formation depends on *tapA* and *epsA-O* expression.

In recent years, there has been an increasing interest in the quorum-sensing signaling molecules related to food spoilage. Various signaling compounds associated with QS, such as AI-2, have been detected in different food systems such as milk (Pinto et al., 2007). Furthermore, studies have shown that QS is important for social behavior of *B. subtilis* and other bacteria (Lombardía et al., 2006). Using *V. harveyi* as a reporter strain for bioluminescence, we were able to track the level of produced AI-2 molecules. We observed an increase in the AI-2 production as a response to lactose in dose dependent manners (**Figure 4**). It has been shown previously that simple dietary sugars can affect QS, specifically production of AI-2 by *Klebsiella pneumoniae* (Zhu et al., 2012). In our study, the cell density of all tested samples was the same at the sampling time, consequently, changes in the AI-2 production is apparently not related to the cell density but to the metabolic state of the bacteria. Thus, our results support previous studies that showed that AI-2-dependent signaling is a reflection of metabolic state of the cell and environmental factors and not cell density (Bassler, 1999; Beeston and Surette, 2002). Previous studies also suggested that activation of QS through LuxS can be regulated in response to sugar metabolism by cyclic AMP receptor protein molecules (Lyell et al., 2013). In *B. subtilis* cells, lactose may affect the energetic metabolic balance in the cell, and through second messengers such as cyclic AMP, or CCP can lead to expression of QS genes such as *luxS*.

The main finding of this study is the apparent link between lactose induced biofilm formation and activation of QS system through increased production of AI-2 molecules in *B. subtilis*. Addition of synthetic precursor for AI-2, DPD, to the media resulted in enhanced bundle formation as well as up-regulation of *tapA* expression (**Figure 5**). Similarly, the direct effect of AI-2 molecules on EPS biosynthesis has been observed previously in *Vibrio cholera*e where the AI-2 molecules up-regulated expression of the EPS biosynthesis genes (Hammer and Bassler, 2003). According to our results, examination of biofilm formation in CDM of the *B. subtilis ΔluxS* mutant resulted in deficiency of biofilm formation (bundle, and colony types) (**Figures 6A** and **7**). These results suggested that QS via LuxS cascade plays an important role in biofilm formation in the presence of lactose. This is consistent with previous research which showed that LuxS is important for *B. subtilis* social behavior (motility and biofilm formation) (Lombardía et al., 2006). Another study showed that blocking the AI-2 pathway, using an AI-2 analog, inhibited biofilm formation by *B. subtilis* (Ren et al., 2002). Similar results were found for *Hafnia alvei*, a food-related bacterium that can be found in dairy products. QS in *H. alvei* is required for differentiation of individual cells into a complex multicellular structure of biofilm (Souza Viana et al., 2009).

**FIGURE 8:**
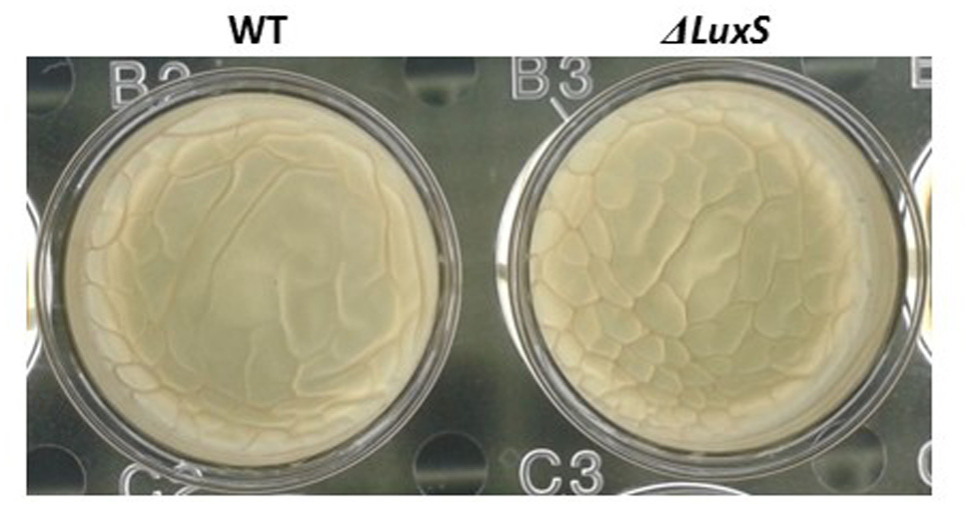
**Pellicle formation by *B. subtilis* in LBGM is not LuxS dependent.** WT and *ΔluxS* cells were used for pellicle formation in LBGM. The pictures were taken using a Zeiss Stemi 2000-C microscope with an axiocamERc 5s camera. Images are representative of two biological repeats.

Interestingly, we observed that the *luxS* mutant strain could form pellicle in biofilm promoting medium LBGM (**Figure 8**). Although, a pellicle formation in LBGM appears to be not LuxS dependent, it seems that in CDM there is a slight induction in pellicle formation in response to lactose (**Figure 6B**). As it was shown recently (Shemesh and Chai, 2013), glycerol and manganese activate KinD-Spo0A pathway for matrix production. In case of lactose, it seems that enhanced production of AI-2 affects not directly on the biofilm formation cascade. This may explain the differences found between phenotypes in CDM supplemented with lactose and in LBGM. Activation of biofilm formation via QS system might be an additional regulatory mechanism which enables fine tuning of the biofilm formation pathway that has been previously described (Shemesh and Chai, 2013).

The LuxS system possesses an inherent metabolic function in the activated methyl cycle; phenotypic defects in *luxS* mutants may not strictly be attributed to AI-2 signaling but possibly to metabolic disturbances. For instance, biofilm defects in a *Lactobacillus rhamnosus luxS* mutant are not restored by AI-2 molecules but rather by the addition of cysteine, indicating a sole metabolic role of LuxS (Lebeer et al., 2007). In order to test whether the deficiency of biofilm formation in the presence of lactose in the mutant strain is due to AI-2 signal molecules or due to metabolic reason, we used DPD for complementation tests. It was shown previously that the synthetic AI-2 precursor (DPD) has the ability for specific AI-2 complementation during biofilm formation by *Streptococcus intermedius* (Ahmed et al., 2008). In the complementation test, we observed restoration of the biofilm phenotype. The *ΔluxS* mutant showed ability for increased bundle formation in media supplemented with lactose and 200 μM of DPD (**Figure 7**), indicating that the abolished biofilm formation is mostly connected to AI-2 and not to LuxS enzyme function.

In overall, results of the present study suggest that QS via LuxS system plays an important role in biofilm formation induced by lactose in *B. subtilis*. As lactose affects activation of LuxS system, it is likely related to activation of Spo0A which leads to biofilm formation through a known pathway of up-regulation of the extracellular matrix operons. Moreover, Spo0A has been shown to be a negative regulator of LuxS system (Lombardía et al., 2006). Additional research on lactose in association with QS will further elucidate the role of QS in biofilm formation of *Bacilli* and the effect of this dairy component on biofilm related gene expression.

## Author Contributions

DD-A together with MS planned the experiments and wrote the original manuscript. DD-A performed the experiments described in the manuscript. DS and YC assisted in planning biofilm experiments as well as revised the manuscript critically for important intellectual content. DD-A, DS, and MS integrated all of the data throughout the study and crafted the final manuscript.

## Conflict of Interest Statement

The authors declare that the research was conducted in the absence of any commercial or financial relationships that could be construed as a potential conflict of interest.
