# Supplementary material for: The LuxS Based Quorum Sensing Governs Lactose Induced Biofilm Formation by Bacillus subtilis
Source: Front Microbiol. 2016 Jan 8;6:1517. doi: 10.3389/fmicb.2015.01517 (PMC4705240; doi:10.3389/fmicb.2015.01517)
Supplement: Supplementary file 1 [file Data_Sheet_1.DOCX]

**Supplementary Material**

**The LuxS based quorum sensing governs lactose induced biofilm formation by *Bacillus subtilis***

D. Duanis-Assaf^1,2^, D. Steinberg^2^, Y. Chai^3^, M. Shemesh^1*^

^1^Department of Food Quality and Safety, Institute for Postharvest Technology and Food Sciences, Agricultural Research Organization (ARO), The Volcani Center.

^2^Biofilm Research Laboratory, Institute of Dental Sciences, Faculty of Dental Medicine, Hebrew University-Hadassah.

^3^Department of Biology, Northeastern University, Boston, Massachusetts 02115, USA

^*^Correspondence

Moshe Shemesh, Department of Food Quality and safety, Agricultural Research Organization, The Volcani Center, Bet Dagan 50250, Israel.

E-mail: [moshesh@agri.gov.il](mailto:moshesh@agri.gov.il)

Phone: 972-39683868; Fax: 972-39604428.

**Supplementary material and methods**

**Growth curve analysis.** An overnight culture of cells was diluted 1:100 (to obtain O.D.­ _(600)_ of 0.07) into LB supplemented with different concentrations of lactose (0-3% w/v). Bacteria were grown in shaking culture 150 rpm, at 37°C. Optical density (O.D.­ _(600)_) of the samples was taken every hour during 8 hours growth.

**Supplementary results**

**Supplementary Figure 1. Growth curve analysis of *B. subtilis* 3610 in the presence of different** **concentrations of lactose**

*B. Subtilis* cells were grown in LB supplemented with 0-3% lactose. Cultures were then incubated at 37 °C and 150 rpm. O.D. results were recorded every hour. The data are displayed as a mean value of results from duplicate experiments.
